# Developing Next-Generation Live Attenuated Vaccines for Porcine Epidemic Diarrhea Using Reverse Genetic Techniques

**DOI:** 10.3390/vaccines12050557

**Published:** 2024-05-19

**Authors:** Ruisong Yu, Shijuan Dong, Bingqing Chen, Fusheng Si, Chunhua Li

**Affiliations:** Institute of Animal Husbandry and Veterinary Medicine, Shanghai Key Laboratory of Agricultural Genetics and Breeding, Shanghai Engineering Research Center of Breeding Pig, Shanghai Academy of Agricultural Sciences (SAAS), Shanghai 201106, China; yuruisong@saas.sh.cn (R.Y.); dsjuan@saas.sh.cn (S.D.); c13919966733@163.com (B.C.)

**Keywords:** PEDV, virulent protein, reverse genetic techniques, live attenuated vaccine, rational design

## Abstract

Porcine epidemic diarrhea virus (PEDV) is the etiology of porcine epidemic diarrhea (PED), a highly contagious digestive disease in pigs and especially in neonatal piglets, in which a mortality rate of up to 100% will be induced. Immunizing pregnant sows remains the most promising and effective strategy for protecting their neonatal offspring from PEDV. Although half a century has passed since its first report in Europe and several prophylactic vaccines (inactivated or live attenuated) have been developed, PED still poses a significant economic concern to the swine industry worldwide. Hence, there is an urgent need for novel vaccines in clinical practice, especially live attenuated vaccines (LAVs) that can induce a strong protective lactogenic immune response in pregnant sows. Reverse genetic techniques provide a robust tool for virological research from the function of viral proteins to the generation of rationally designed vaccines. In this review, after systematically summarizing the research progress on virulence-related viral proteins, we reviewed reverse genetics techniques for PEDV and their application in the development of PED LAVs. Then, we probed into the potential methods for generating safe, effective, and genetically stable PED LAV candidates, aiming to provide new ideas for the rational design of PED LAVs.

## 1. Introduction

Porcine epidemic diarrhea (PED) is a highly contagious intestinal disease in swine caused by porcine epidemic diarrhea virus (PEDV), with typical clinical symptoms including severe diarrhea, vomiting, and dehydration, often leading to up to 100% mortality in suckling piglets [1]. PEDV is mainly transmitted through the fecal–oral route, but recent reports have also suggested potential transmission through the fecal–nasal route or aerosol means [2,3]. PED was first reported in England in the 1970s, and it subsequently spread to most of the pig-rearing countries, including Asia and, most recently, North America, resulting in serious problems and substantial economic losses to the world’s swine industry [4,5,6]. Vaccinating pregnant sows used to be the main strategy for the prevention and control of classical PEDV strains (genotype 1, G1). However, since the end of 2010, currently available PED vaccines have not been able to offer sufficient protection for piglets against infection by the highly pathogenic PEDV variants (genotype 2, G2) that have emerged due to cumulative mutations and recombination events [7,8,9].

**Figure 1:**
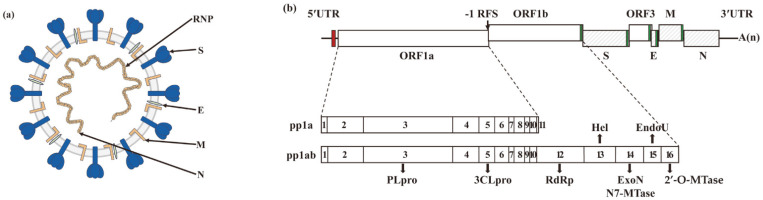
Schematic representation of the PEDV virion (**a**) and its genome organization (**b**). The red and green bars in (**b**) represent the leader transcriptional regulatory sequence (TRS) and body TRS, respectively. The expression of ORF1a and ORF1b yields two known polyproteins (pp1a and pp1ab) through a -1 programmed reading frameshift (RFS). These polyproteins are then processed into 16 distinct nonstructural proteins (nsp1–16), with the numbers for pp1a and pplab indicating nsp1–16. Abbreviations: RNP, ribonucleoprotein; S, spike; ORF3, open reading frame (ORF) 3; E, envelope; M, membrane; N, nucleocapsid; 5′UTR, 5′ untranslated region; 3′UTR, 3′ untranslated region; A(n), polyadenylated tail; PLpro, papain-like cysteine protease; 3CLpro, 3C-like cysteine protease; RdRp, RNA-dependent RNA polymerase; Hel, helicase; ExoN, 3′-5′ exonuclease; N7-MTase, N7-methyltransferase; EndoU, endoribonuclease; 2′-O-MTase, ribose-2′-O-methyltransferase. Adapted from Niu et al. [13] and Jang et al. [6].

PEDV is a member of the genus *Alphacoronavirus* (α-CoV) in the family of *Coronaviridae* of the order *Nidoviruales*. The single-stranded positive-sense RNA genome of PEDV, which is approximately 28 kb in size, encodes four structural proteins, namely spike (S), envelop (E), membrane (M), and nucleocapsid (N) proteins, along with sixteen nonstructural proteins (nsp1–nsp16) and an accessory protein, ORF3 [10]. The viral structural proteins come together to form progeny virions, while the nonstructural proteins constitute the replication/transcription complex (RTC) that is primarily responsible for viral replication, transcription, and translation (Figure 1) [11]. The accessory protein ORF3 plays a crucial role in PEDV infection, particularly in its interaction with host cells [12].

Innate immunity plays a crucial role in host defense against invading pathogens. The activated innate immune system produces interferons (IFNs), proinflammatory cytokines/chemokines, and other immune factors which perform antiviral functions to eliminate invading viruses [14,15]. Correspondingly, viruses have developed diverse strategies to evade hosts’ innate antiviral responses to benefit their proliferation during their coevolution with hosts [16,17,18]. PEDV can counteract a host’s innate immune responses through various mechanisms to facilitate its replication and pathogenesis. For instance, PEDV infection activates mitogen-activated protein kinase (MAPK) cascade pathways to promote its infection [19,20]. PEDV also activates the phosphatidylinositol-3-kinase (PI3K)/protein kinase B (PKB/AKT) pathway during the viral entry and replication stages, while its effect on PEDV proliferation has yet to be fully deciphered [21,22]. Kong et al. found that inhibiting the PI3K/AKT/GSK-3α/β (glycogen synthase kinase 3, GSK-3) pathway via blocking PI3K activation or GSK-3 phosphorylation enhanced PEDV replication in Vero cells, suggesting the PI3K/AKT/GSK-3α/β pathway activated by PEDV infection functioned to inhibit PEDV replication [21]. However, Yang et al. found that inhibition of the PI3K/AKT/mTOR pathway with the corresponding inhibitors suppressed PEDV infection in both Vero-E6 and intestinal porcine epithelial (IPEC-J2) cells [23]. Similarly, it has also been found that suppressing the PEDV-induced PI3K/AKT pathway with the PI3K inhibitor inhibited PEDV entry into Vero-E6 cells via micropinocytosis, suggesting that PEDV might use PI3K/AKT-dependent micropinocytosis to enter cells [22]. PEDV infection up-regulates the expression of death receptor 5 (DR5) in both Vero cells and piglets to facilitate virus production by activating viral-induced caspase-8-dependent apoptosis and facilitating viral entry [24]. PEDV infection induces endoplasmic reticulum (ER) stress through activating unfolded protein response (UPR) signaling pathways in Vero cells [25]. Furthermore, PEDV can temporally modulate ER stress to benefit its replication by attenuated host UPR signaling pathways at early time points (0–6 h post infection), then up-regulate them dramatically from 12 h to 48 h [25]. PEDV infection induces nuclear factor-κB (NF-κB) activation through Toll-like receptor (TLR)2, TLR3, and TLR9, but not retinoic acid inducible gene I (RIG-I)-like receptor signaling pathways in porcine intestinal epithelial cells [26]. In addition, PEDV infection promotes the accumulation of lipid droplets (LDs) by activating the NF-κB signaling pathway to promote its replication. The inhibition of LD accumulation and the NF-κB signaling pathway with quercetin significantly inhibited PEDV replication in vitro and in vivo [27]. Moreover, PEDV infection induces pyroptosis in Vero cells, and inhibiting pyroptosis suppresses the proliferation of PEDV [28]. PEDV could induce apoptosis in infected cells both in vitro and in vivo to enhance its proliferation [29,30]. PEDV infection induces autophagy to facilitate its replication in Vero cells via reactive oxygen species (ROS)-dependent endoplasmic reticulum (ER) stress through activating the protein kinase RNA (PRKR)-like endoplasmic reticulum kinase (PERK) and inositol-requiring enzyme-1 (IRE1) pathways [31]. It has also been found that inducing ferroptosis with activators could inhibit the replication of PEDV in Vero cells [32,33], suggesting that the ferroptosis pathway also influences PEDV infection. PEDV not only fails to induce IFN-β expression, but also inhibits dsRNA-mediated IFN-β production in IECs [34]. Mechanistically, PEDV could inhibit dsRNA-induced IFN-β production in porcine intestinal epithelial cells by blocking the activation of IFN-β promoter stimulator 1 (IPS-1) in the RIG-I-mediated pathway [34]. Meanwhile, PEDV can subvert the type I IFN response by inducing STAT1 degradation [35]. The structural and nonstructural proteins encoded by the PEDV genome are the main executors of regulating cellular signaling pathways and counteracting innate immune responses to achieve a productive viral infection. Therefore, deciphering the intricate interaction mechanisms between PEDV and the host and identifying the key viral virulent proteins will play a crucial role in the prevention and control of PED epidemics.

As piglets are born with agammaglobulinemia and are immunodeficient until weaning [36,37], newborn piglets cannot promptly mount protective immunity against various infections and rely exclusively on colostrum and milk from immunized sows to combat infections during their early lives [38]. Thus, an ideal PED vaccine should induce efficient maternal antibodies in pregnant sows, then transmit these antibodies to neonatal piglets in the form of colostrum or milk and protect them from PEDV. More and more evidence demonstrates that the replication capability of a vaccine strain in sow enterocytes is critical for triggering the gut–mammary gland–secretory IgA (sIgA) axis and inducing sufficient maternal immunity to protect piglets from virulent PEDV infection [39,40,41]. Compared with the killed or subunit vaccines administrated via intramuscular injection, live attenuated vaccines (LAVs) administrated orally or intranasally can induce more potent and long-lasting maternal immunity, making them the most ideal vaccine for the prevention and control of PED [38]. The effectiveness and safety of commercial PED LAVs are two key factors influencing their clinical application. On the one hand, LAV strains obtained through serial passaging in cultured cells often fail to induce sufficient maternal immunity due to their reduced replication capability and immunogenicity in natural hosts (pregnant sows). One the other hand, the accumulation of compensatory mutations or recombination with field strains during their clinical application can lead to the virulence reversion of LAV strains [42,43,44]. Therefore, to develop a safe and effective PED LAV, it is crucial to maximally preserve the viral antigenicity and replication capability in the sow intestine while reducing its virulence. In this context, the establishment of reverse genetic systems (RGSs) for PEDV provides an essential tool for purposefully manipulating the viral genome to generate safe and effective PED LAV candidates. Compared with commercial LAVs generated by the repeated passage of virulent PEDVs in vitro, RGS-generated PED LAVs are rationally designed, with advantages such as (1) genetic stability by introducing attenuation mutations at multiple sites and/or though different mechanisms into the virus genome to resist the reversion to virulence caused by reverse/complementary mutations or recombination events; (2) high immunogenicity by rationally introducing attenuation mutations into antigenic proteins or codon (de)optimizing strategies to maximally keep viral antigenicity; and (3) strong flexibility as the attenuation strategy is clear and rationally designed, which can easily be used for new emerging variant strains or even other coronaviruses. This review summarizes the recent advances in virulence-related viral proteins, RGSs for PEDV, and their application in generating PED LAV candidates. Finally, a brief outlook is provided on the development of next-generation PED LAV candidates by using RGSs.

## 2. PEDV Proteins as Virulence Factors

To counteract the innate responses induced by viral genomic or subgenomic RNA, the PEDV genome encodes various structural and nonstructural proteins to regulate host cell protein expression, interfere with host innate immune response signaling pathways, and regulate the cell cycle, thereby creating a favorable environment for viral proliferation and diffusion. Multiple viral proteins have been shown to be virulence factors of PEDV. For instance, five nonstructural proteins (nsp1, nsp3, nsp14, nsp15, and nsp16), three structural proteins (E, M, and N), and the only known accessory protein (ORF3) encoded by the PEDV genome can antagonize both type I and type III interferon (IFN) production and signaling [45,46], although their mechanisms of action vary. Exogenous expression studies indicated that PEDV S1 induced the highest level of apoptosis [47]. The functions and known key motifs of PEDV proteins associated with virulence or pathogenicity are listed in Table 1.

### 2.1. Structural Proteins

In addition to being the components of viral particles, all the PEDV structural proteins have been shown to regulate cell processes, such apoptosis, proinflammatory cytokine production, and the IFN signaling pathway, to facilitate virus proliferation. The E, M, and N proteins are all potent IFN antagonists [45,46].

#### 2.1.1. S Protein

In order to enter host cells for replication, PEDV binds to cell receptors via the S1 subunit of the S protein, and then fuses with the host cell membrane through its S2 subunit. As the largest glycoprotein on the viral surface, the S protein is the main inducer of neutralizing antibodies against PEDV. Hence, in order to evade the host’s humoral immunity and achieve the repeated infection of previously exposed pigs, PEDV variants accumulate a large number of mutations in the S protein [48,114]. Accordingly, the S protein is considered one of the determinants for the high pathogenicity of the emerging PEDV variants. To verify this hypothesis, researchers have swapped the S gene of virulent and attenuated PEDV strains using RGSs and found that the S gene significantly affects the pathogenesis of recombinant PEDVs [114,115]. Specially, recombinant PEDV with the RGS-generated deletion of 197 amino acids (34–230) or the spontaneous deletion of 205 amino acids (23–229) in the N-terminal domain of the S1 subunit showed reduced virulence in piglets compared to their corresponding parental strains [116,117]. Similarly, substituting the S1 subunit of PEDV CV777 with the corresponding part from the highly virulent Japanese strain can significantly increase the pathogenicity of PEDV variants in Gn neonatal piglets [49], underscoring the critical role of the S1 subunit as a determinant of PEDV virulence and immunogenicity. Likewise, the C-terminal domain also influences PEDV virulence, as deleting the two C-terminal intracellular sorting motifs, YEVF and KVHVQ, of the S protein using an RGS significantly reduces the virulence of the recombinant PEDV in piglets [50,51]. However, these studies also found that although the S protein or its S1 subunit contributes to the observed differences in virulence between attenuated and virulent PEDV strains, they are not the only virulence determinants.

Although there is still considerable controversy about PEDV receptors, several cellular surface proteins have been identified as regulators of PEDV infection by interacting with the PEDV S protein. The S1 subunits of PEDVs are supposed to bind to porcine aminopeptidase N (pAPN) and sialic acid glycan via their N-terminal domain (NTD) and the C-terminal domain (CTD), respectively [118,119]. Under in vitro and in vivo conditions, the PEDV S1 subunit can bind to transferrin receptor 1 (TfR1) on the cell surface, inducing endosomal vesicle formation to facilitate PEDV entry [120]. In contrast, the binding of PEDV S1 to the DnaJ heat shock protein family (Hsp40) member A3 (DNAJA3) protein on the cell surface via its central part (aa 379–479) interferes with the attachment of PEDV to host cells [121]. In addition, during PEDV infection, the S1 subunit binds to the epidermal growth factor receptor (EGFR) and triggers the EGFR signaling pathway, ultimately inhibiting the activity of the sodium–hydrogen ion transporter, Na^+^/H^+^ exchanger (NHE3) [52]. Mechanistically, during PEDV infection, the S1-EGFR-ERK (extracellular-signal-related kinase) signaling pathway is activated to reduce the expression and mobility of NHE3 on the cell surface, impairing Na^+^ absorption by the intestine and resulting in diarrhea in piglets. Furthermore, the interaction of the S1 subunit with the EGFR also impairs type I IFN signaling by activating the signal transducers and activators of transcription 3 (STAT3) [53]. Lastly, as the potent apoptotic inducer of PEDV, more research is warranted to decipher how S1 regulates cell fate to facilitate viral proliferation [47]. Moreover, to generate a rationally designed PEDV LAV candidate using RGSs by mutating the S gene while keeping its antigenicity, it is necessary to clarify the exact region or the key amino acids of the S protein that interact with these cellular proteins.

#### 2.1.2. E Protein

The small envelope protein is involved in the assembly and budding of PEDV. The subcellular localization of exogenously expressed E protein is the ER, although a little has been detected in the nucleus. The expression of the E protein does not impact intestinal epithelia cell (IEC) proliferation and the cell cycle, but induces ER stress via up-regulating glucose-regulated protein 78 (GRP78) and activates the NF-κB signaling pathway to up-regulate the level of the proinflammatory neutrophil chemotactic factor IL-8 and the anti-apoptotic molecule Bcl-2 [55]. The exogenous expression of the E protein can also suppress the production and signaling of both type I and type III IFNs induced by IFN agonists [45,46]. Zheng et al. studied the mechanism by which the PEDV E protein antagonized the type I IFN signaling pathway and found that the E protein triggered the PERK/eIF2α (eukaryotic initiation factor 2α)-pathway-mediated ER stress response, attenuating the expression of key proteins involved in the RIG-I-MAVS-IRF3 signaling pathway, rather than their transcription [56,58]. Additionally, the E protein can also bind to the IFN regulatory factor (IRF3) and inhibit its nuclear translocation [56]. In addition, the E protein has been shown to inhibit the transcription of swine leukocyte antigen II DR (SLA-DR) in PEDV-infected dendritic cells, impairing the antigen presentation, CD4+ T cell activation, and subsequent humoral immunity response [59]. Accordingly, the host cells inhibit the proliferation of PEDV by the karyopherin α2 (KPNA2)-mediated autophagic degradation of the E protein [122], while the detailed mechanism of the interaction between E and KPNA2 needs further research. The transmembrane domain (TM) of the E protein is critical in its function. For instance, A PEDV variant obtained by serial passage in vitro, with a deletion of ^16^LWLFV^20^ and an L^25^ to P^25^ mutation in the TM of the E protein, can induce more potent ER stress, cell apoptosis, and higher levels of IL-6 and IL-8 [54]. Similarly, deletion of the 7-amino acid (aa) (^23^FLLIISI^29^) in the TM domain of the E protein using an RGS enables the recombinant PEDV to induce higher levels of both type I and type III IFNs, and is significantly attenuated in vivo [57].

#### 2.1.3. M Protein

Among the structural proteins of PEDV, the M protein is the most abundant in virions. In addition to participating in virus assembly and morphogenesis, the M protein also regulates the cell cycle and host’s innate immune responses through interactions with cellular proteins. Overexpression of the M protein promotes the proliferation of PEDV [60]. Exogenous expression of the M protein in IEC down-regulates the transcription of cyclin A, leading to cell cycle arrest at the S phase, while having no effect on the expression of the ER stress-, inflammatory-, and apoptosis-related molecules [61]. The M protein can suppress the expression of both type I and type II IFNs, but through different mechanisms [45,46]. The PEDV M protein suppresses the type I IFN signaling pathway by binding and incapacitating IFN regulatory factor 7 (IRF7) via the 1–55 aa region, inhibiting the phosphorylation and dimerization of IRF7 [60]. In contrast, the M protein antagonizes type III IFN production by interfering with IRF1 promoter activity [46]. Our proteomic studies on M interacting proteins have identified multiple host proteins involved in immune response, apoptosis, and cell cycle pathways, among which S100 calcium-binding protein A11 (S100A11) and peptidyl-prolyl cis-trans isomerase D (PPID) were shown to suppress PEDV proliferation [123,124]. Furthermore, the M protein was shown to co-localize with the expression of heat shock protein 70 (HSP70) and increased HSP70 expression in both overexpression and virus infection conditions, enhancing PEDV replication [62]. However, how HSP70 interacts with the M protein and promotes PEDV replication remains to be elucidated.

#### 2.1.4. N Protein

The coronavirus N protein contains multifunctional domains and is involved in virus replication, assembly, and budding, as well as in modulating host cell response and fate during the virus’s productive proliferation [125]. As one of the vital virulence proteins, the PEDV N protein has been shown to arrest the cell cycle, regulate proinflammatory cytokine production, and antagonize IFN synthesis and signaling [63,68].

The PEDV N protein localizes in the endoplasmic reticulum (ER), induces ER stress via up-regulating GRP78, and activates NF-κB via the TLR2 signaling pathway to up-regulate proinflammatory cytokine expression [26,63]. The central region of the N protein (136–289 aa) was most potent in the activation of NF-κB. Huan et al. showed that the PEDV N protein not only bound to CCAAT/enhance-binding protein (C/EBP)-β to increase the transcription of the high mobility group box 1 protein (HMGB1), but also increased the acetylation/release of HMGB1 as well as the levels of proinflammatory cytokines [126]. In addition, the N protein arrests the cell cycle of both intestinal epithelial cells (IECs) and Vero cells in the S phase via interacting with p53, facilitating PEDV replication [63,65]. Mechanistically, the N protein interacts with p53 in the nucleus via the N^S171–N194^ motif, maintaining a high level of p53 and activating the p53-DREAM signaling pathway to inhibit the expression of cyclin A, and subsequently inducing S phase arrest [65]. A subsequent proteomic study showed that 60S ribosomal protein L18 (RPL18) was significantly increased in S phase arrested cells induced by N protein expression or PEDV infection [127]. In contrast, a recent study found that the N protein virus bound to constitutively photomorphogenic 1 (COP1) via its N-terminal domain (1–124 aa) and inhibited COP1 degradation, thereby promoting the COP1-mediated ubiquitination and degradation of p53, abolishing the antiviral activity of p53 [66]. To elucidate the role of p53 in PEDV infection, the interaction between p53 and the N protein deserves further investigation.

The PEDV N protein antagonizes type I IFN production and signaling in HEK-293T cells by interfering with the interaction of TANK (TRAF family member associated NF-κB activator)-binding kinase 1 (TBK1) with IRF3, inhibiting IRF3 phosphorylation [68]. However, in another study by Shan et al., it was found that the PEDV N protein antagonizes type III IFN production by inhibiting the nuclear translocation of NF-κB in IPEC-J2 cells, having no effect on the levels of type I or type II IFNs [67]. PEDV antagonizes type I IFN signaling in IPEC-J2 cells by interrupting the phosphorylation of signal transducer and activator of transcription 1 (STAT1) and promoting STAT1 degradation via the ubiquitin-proteasome system [35]. The PEDV-N inhibits the production of IFNs in IPEC-J2 cells and inhibits the type I IFN-mediated JAK1/STAT1 signaling pathway via inducing STAT1 degradation and interrupting STAT1 phosphorylation [69]. The PEDV N protein interacts directly with the host transcription factor Sp1 in the nucleus, interfering with its binding to the promoter region, thereby inhibiting its transcriptional activity for histone deacetylase 1 (HDAC1) expression, leading to decreased expression of ISGs and facilitating virus replication [128]. Subsequent research revealed that the PEDV N protein inhibits STAT1 phosphorylation and nuclear localization by inducing STAT1 acetylation via the down-regulation of HDAC, which in turn, dampens the host IFN-λ signaling activation [129]. Li et al. found that the PEDV N protein interacted with the Really Interesting New Gene (RING) domain of TRIM28 (a member of the tripartite motif proteins), and the co-existence of TIM28 and PEDV N promoted mitophagy, significantly inhibiting the IFN-β-JAK/STAT1 signaling pathway in host cells, thus facilitating virus replication [130]. However, more studies are needed to address the molecular mechanism by which the PEDV N protein up-regulates the expression of TRIM28 and how TRIM28-mediated mitophagy down-regulates the expression of STAT1. Additionally, the PEDV N protein also inhibits the expression of angiotensin converting enzyme 2 (ACE2), which otherwise promotes the expression of ISGs by activating the JAK1/STAT1 pathway and promoting STAT1 phosphorylation, thus relieving PEDV-induced inflammation and cell injury [69].

Overexpression of the N protein can also increase the intracellular content of viral RNA and promote PEDV replication [70]. As a viral suppressor of RNA silencing, the PEDV N protein suppresses short hairpin RNA-induced RNA interference of the host cell to promote viral replication, potentially affecting viral pathogenesis [131]. Research has also shown that the PEDV N protein can interact with the zinc-finger antiviral protein (ZAP) in host cells to antagonize its antiviral activity [132]. Mechanistically, the PEDV N protein suppresses the activation of ZAP by its essential cofactor, tripartite motif-containing protein 25 (TRIM25), in which the N protein interacts with both ZAP and TRIM25 through its N-terminal domain and C-terminal domain, respectively [133]. Additionally, the interaction with the N protein also suppresses E3 ligase activity in TRIM25, which is required for the antiviral activity of ZAP. Moreover, the PEDV N protein interacts with and prevents the proteolytic cleavage of the host nucleolar phosphoprotein nucleophosmin (NAM1) in the nucleolus, enhancing host cell survival and prompting PEDV proliferation [64]. Furthermore, the PEDV N protein could interact with heterogeneous nuclear ribonucleoprotein A1 (hnRNP A1), a cellular protein participating in pre-mRNA splicing in the nucleus and translation regulation in the cytoplasm, to promote PEDV replication [134]. As the PEDV N protein plays an important role in counteracting the host’s innate immune responses via multiple methods to promote viral proliferation, it is a potential target to inhibit virus replication by the host’s innate immunity. Recently, Shan’s group revealed that host factors, such as early growth response gene 1 (EGR1), RALY, PBPC4, PTBP1, and RBBM14, could suppress PEDV replication by targeting and degrading the viral N protein through the selective autophagosome pathway and IFN signaling pathway [135,136,137,138].

In addition, the PEDV nucleocapsid protein also interacts with the membrane-bound cytoskeleton linker protein Ezrin and inhibits Ezrin phosphorylation, decreasing the level and activity of the Na^+^/H^+^ exchanger 3 (NHE3) protein on the cell surface, thereby decreasing Na^+^ absorption by the intestine and leading to diarrhea in piglets [71]. Meanwhile, Jaru-Ampornpan et al. found that the cleavable N protein by PEDV 3C-like protease contributes to the viral adaptation to cell culture. This finding was further confirmed by the growth characteristics of recombinant PEDV with the mutated N protein generated using RGS [72].

### 2.2. Accessory Protein

ORF3, the only known accessory protein of PEDV, has ion channel activity and can regulate virus replication and virulence through various mechanisms. These include trapping cells in this S phase, promoting intracellular vesicle formation, inhibiting cell apoptosis, antagonizing INF activity, and promoting autophagy [30,73,74,75,77,139]. Despite the use of sensitive antibodies and assays, no ORF3 protein could be detected in highly purified PEDV particles, indicating that the protein is not a virion structural component [140].

The PEDV ORF3 protein functions as an ion channel and enhances virus release during infection [73]. It is mainly localized in the ER and triggers ER stress by up-regulating GRP78 protein expression and activating the PERK-eIF2α signaling pathway [74]. Our study shows that the ORF3 protein utilizes the exocytic pathway via its C-terminal YxxØ motif to accumulate in the Golgi area of both the infected and transfected cells, as well as on the surface of PEDV-infected cells [140]. Heterogeneous expression of the ORF3 protein has been shown to prolong the S phase of host cells, facilitate the formation of vesicular structures, and promote the proliferation of attenuated PEDV rather than its virulent counterpart [75]. In addition, ORF3 induces ER stress-dependent autophagy by promoting the conversion of LC3-I to LC3-II, without influencing apoptosis [74]. Our finding indicates that both the intact ORF3 and its naturally truncated form can inhibit apoptosis to facilitate virus replication [30]. The conflicting results observed in different studies may be attributed to the use of different experiment models. For instance, our study involved cells infected with recombinant PEDV with or without ORF3, while another study utilized ORF3-expressing cells. This suggests that the functions of viral proteins should be verified, at the very least, in a viral infection environment.

Previous studies have shown that PEDV ORF3, as a viral virulence factor, can suppress the activation of NF-κB and IRF1 promoters, thereby inhibiting the production of type I and type III IFNs, respectively [46,78]. In addition, ORF3 serves as a potent suppressor of proinflammatory cytokines. Mechanistically, ORF3 blocks the phosphorylation of inhibitory kappa B alpha (IκBα) and interferes with the expression of NF-κB p65 as well as its phosphorylation and nuclear translocation, ultimately leading to the suppression of proinflammatory cytokine (IL-6 and IL-8) production [76]. An interactome study demonstrated that ORF3 was involved in endo-lysosomal and innate immune signaling pathways [141]. Specifically, the interaction between ORF3 with the inhibitor of nuclear factor kappa-B kinase subunit beta (IKBKB) and the vacuolar protein-sorting-associated protein 36 (VPS36) was verified. The VPS36 interacts with and targets ORF3 for degradation, thereby suppressing PEDV replication [141]. Subsequent studies have shown that the binding of ORF3 to IKBKB does not interfere with the binding of NEMO to IKBKB, but increases IKBKB-mediated NF-κB promoter activity [77]. In addition, ORF3 significantly reduces the transcription of type I IFNs mediated by IKBKB, but substantially increases the production of type I IFNs mediated by IKBKB, the mechanism of which is unknown [77]. Although it does not directly interact with proteins in the RIG-I-like receptor (RLR) signaling pathway, the ORF3 protein not only inhibits the phosphorylation and nuclear transportation of IRF3, but also specially reduces the expression of these proteins to antagonize the production of type I IFNs and ISGs [142]. In addition, similar to the E protein, the ORF3 protein can also inhibit the transcription of SLA-DR, potentially affecting the host’s humoral immunity [59]. Additionally, both the intact and naturally truncated ORF3 proteins can directly bind to the S protein, while their effects on PEDV replication and pathogenesis are unknown [139]. These complex interactions between ORF3 and the innate immune signaling pathway are mainly based on in vitro overexpression studies. In order to elucidate the effects of ORF3 on PEDV proliferation, further studies are needed on recombinant PEDV with mutated ORF3 or without ORF3 generated using RGSs.

ORF3 is dispensable for virus growth in vitro, and the truncation or deletion of ORF3 usually leads to reduced virulence in natural hosts [143,144]. Although ORF3 can increase virus infection and lesions in the intestinal tract of swine [114], not all truncated forms of ORF3 are associated with reduced viral pathogenicity [145,146,147]. Several studies have also confirmed that ORF3 is not associated with the pathogenicity of PEDVs [148,149]. Direct evidence supporting ORF3 as a PEDV virulence factor comes from studies with recombinant PEDV constructed via a reverse genetics system [150,151,152]. These studies demonstrated that pigs infected with a recombinant PEDV lacking ORF3 [150,152] or with a natural truncated form of ORF3 [151] had lower clinical scores than those infected with the wildtype PEDV strain. Moreover, both recombinant PEDVs were still able to efficiently transmit from infected pigs to uninfected pigs via indirect or direct contact, leading to disease outcomes. Collectively, these findings suggest that the impact of PEDV ORF3 on viral pathogenicity is modest. 

However, the current evidence does not fully elucidate how ORF3 governs virus pathogenesis and replication in vitro and in vivo. It is speculated that the effect of ORF3 may largely depend on the PEDV strain, ORF3 itself, and the cell line used in each study. Thus, future research should focus on identifying the key amino acids or functional domains by which ORF3 affects viral virulence in vivo and cell adaptability in vitro, which are crucial for the generation of PED LAV candidates by modifying the ORF3 gene.

### 2.3. Nonstructural Proteins

In addition to being responsible for viral transcription and replication, coronavirus nsps also participate in antagonizing the host’s innate immunity against virus infections. The multiple nsps of PEDV have the function of regulating cell metabolism, inhibiting the IFN signaling pathway to favor viral RNA synthesis, structural/nonstructural protein expression, and viral assembly and release [45,46]. Unveiling the roles of various nsps in evading the host’s innate immune response and the pathogenesis of PEDV, and identifying the key amino acids involved, will guide the rational design of PED LAV candidates.

#### 2.3.1. Nsp1

Among the nsps encoded by PEDV, nsp1 is one of the critical antagonists of proinflammatory cytokines and the IFN signaling pathway, acting through various pathways [45,46,78]. Viral infections typically activate NF-κB, inducing the production of IFNs and proinflammatory cytokines. The nsp1 protein has been shown to block the transcription of type I IFNs and proinflammatory cytokines in the nucleus by interfering with the NF-κB signaling pathway in the cytoplasm, in which the nsp1 protein reduces the phosphorylation and degradation of IκBα, thereby interfering with the release and entry of p65 into the nucleus [78]. PEDV nsp1 also promotes the proteasome-dependent degradation of CREB-binding protein (CBP) in the nucleus, disrupting the assembly of IFN regulatory factor 3 (IRF3) and CREB-binding protein (CBP) by the proteasomal degradation of CBP into the enhanceosome, thereby ultimately inhibiting type I IFN expression [45]. Furthermore, nsp1 suppresses IRF1-mediated type III IFN activity by blocking the nuclear translocation of interferon regulator factor 1 (IRF1) and reducing the number of peroxisomes [46]. The suppression of innate immune responses by PEDV nsp1 relies on its highly conserved residues [46,78], particularly residues N93 and N95, which are crucial for type I and type III IFN suppression [45,46]. This was confirmed by a subsequent study on piglets infected with recombinant PEDV carrying nsp1 mutations generated using an RGS [153]. Compared with the parental strain, the PEDV N93/95A mutant replicated to a significantly lower infectious titer, triggered a stronger type I and type III IFN response, and was more sensitive to IFN treatment in vitro. Additionally, the mutant PEDV was attenuated and induced 100% protection against severe diarrhea and death post-challenge in neonatal gnotobiotic (Gn) pigs [153]. Similarly, compared with the parental strain, the PEDV nsp1 F44A mutant induced higher mRNA levels of IFN-β, IFN-λ1, and ISG54 in LLC-PK cells, resulting in a lower viral titer [80]. However, the pathogenicity and the immunogenicity of the F44A mutant need to be evaluated in pigs. PEDV nsp1 inhibits the type I IFN signaling pathway by down-regulating the phosphorylation of STAT1 at S727, and inhibits cell proliferation by arresting the cell cycle at the G0/G1 phase [79]. Moreover, PEDV nsp1 reduces complement component 3 (C3) expression through inhibiting CCAAT/enhancer-binding protein β (C/EBP-β) phosphorylation via V^50^, which is crucial in preventing viral infection [81]. And mutation of the residual V50 in nsp1 can attenuate the immune evasion capability of the recombinant PEDV constructed using an RGS. Furthermore, it has been shown that PEDV nsp1 plays a role in triggering host translation shutoff, and the motif comprising amino acids 92–96 is necessary for this inhibition [82]. Whether the mutation of this specific motif of nsp1 could attenuate PEDV as found for the transmissible gastroenteritis virus (TGEV) warrants further studies [82].

#### 2.3.2. Nsp2

Nsp2 acts through a novel mechanism to antagonize the host’s antiviral response and facilitate PEDV replication. Nsp2 interacts with and targets the F-box and WD repeat domain-containing 7 protein (FBXW7, a subunit of the SCF-type E3-ubiquitin ligase complex that marks its substrate for proteasomal degradation) for degradation through the K48-linked ubiquitin-proteasome-mediated pathway. This leads to the decrease in the expression of RIG-I and TBK1, as well as the induction of ISGs [84]. Consistent with in vitro studies, decreased FBXW7 expression was also observed in different intestinal tissues from PEDV-infected specific-pathogen-free (SPF) piglets. Additionally, PEDV nsp2 induces autophagy and recruits the selective autophagic receptor NBR1 to facilitate the ubiquitination of TBK1 at K48 and its subsequent degradation via the autophagosome pathway, thereby inhibiting the host’s innate antiviral response [83]. Although nsp2 is dispensable for PEDV infection, its deficiency impairs virus proliferation in vitro and in vivo. The entire deletion of nsp2 using an RGS results in the loss of pathogenicity of a highly virulent PEDV in neonatal piglets, leading to a significant increase in IFN and ISG levels in intestinal tissues [83]. This suggests that the deletion of nsp2 is a potent means of generating PED LAV candidates; however, more studies should be conducted to verify that nsp2 is non-essential for the proliferation of other PEDV isolates, and the immunogenicity of nsp2-deficient PEDV should be validated in pregnant sows.

#### 2.3.3. Nsp3

Nsp3 is the largest nsp of PEDV and is responsible for the cleavage of the polymerase protein (pp1a and pp1ab) at the nsp1/nsp2 and nsp2/nsp3 sites. PEDV nsp3 contains two papain-like protease domains (PLP1 and PLP2), both of which have been shown to act as type I IFN antagonists. PLP1 acts as a negative regulator of IFN-β and functions to augment PEDV infection by interacting with poly(C) binding protein 2 (PCBP2), which is involved in maintaining mRNA stability and regulating translation and cellular antiviral responses [86]. Meanwhile, PLP2 acts as a viral deubiquitinase (DUB) to suppresses IFN-β production by removing ubiquitinated conjugates from RIG-I and STING (stimulator of interferon genes), thereby circumventing the activation of the RIG-I and STING-mediated signaling pathway [85]. The IFN antagonistic activity of PLP2 depends on the intact catalytic sites at C1729, H1888, and D1901. In addition, compared with their respective parental strains, the nsp3 of the attenuated vaccine strains of PEDV CV777, and DR13 contain 12 and 21 sense mutations, respectively. Both attenuated strains have identical 8-aa deletions [154]. Although the impact of these mutations on viral virulence needs further verification, mutations of nsp3 can be applied to the rational design of PED LAV candidates.

#### 2.3.4. Nsp4

Interestingly, PEDV nsp4 could up-regulate the expression of proinflammatory cytokines and chemokines (e.g., IL-1α, IL-1β, TNF-α, and CCL-5) via the NF-κB pathway and inhibiting PEDV replication in vitro [87]. This suggests that nsp4 might provide a suitable environment for long-term virus proliferation by regulating the production of proinflammatory cytokines and chemokines induced by PEDV infection.

#### 2.3.5. Nsp5

The coronavirus nsp5 gene encodes the viral main protein protease, the 3C-like protease, which is responsible for the post-translational processing of pp1a/pp1ab together with the nsp3 protein [154]. PEDV nsp5 antagonizes the host’s innate immune responses in multiple ways. Firstly, Nsp5 can interact with and activate AKT phosphorylation to activate the PI3K/AKT/mTOR pathway, facilitating PEDV infection [23]. Secondly, PEDV nsp5 induces apoptosis through the mitochondrial antiviral signaling protein (MAVS) signaling pathway and can synergistically promote MAVS-mediated apoptosis in a protease activity-dependent manner [89]. Thirdly, PEDV Nsp5 can antagonize porcine gasdermin D (pGSDMD)-mediated pyroptosis by cleaving pGSDMD at the Q193-G194 junction, thereby facilitating viral replication during the initial period, which is an important strategy for sustaining infection [90]. Fourthly, nsp5 can cleave multiple key effectors in the RIG-I signaling pathway to antagonize type I IFN production and signaling [92]. For instance, nsp5 cleaves the NF-κB essential modulator (NEMO) at glutamine 231 (Q231), depending on its protease activity [91]. Fifthly, nsp5 impairs porcine DNA polymerase delta interacting protein 3 (POLDIP3)-mediated antiviral effects by cleaving POLDIP3 at Q176 via its 3C-like protease activity in a manner independent of the proteasome and cellular autophagy pathways, ensuring efficient PEDV infection [93]. Sixthly, nsp5 indirectly induces the cleavage of lysophospholipid acyltransferases (LPACT3), which play an important role in lipid absorption in the intestines, potentially triggering gastrointestinal symptoms in PEDV infection [88]. In addition, the nsp5-induced LPCAT3 cleavage induces ER stress in IPEC-J2 cells. Seventhly, nsp5 can antagonize the host’s antiviral capability by cleaving histone deacetylase 6 (HDAC6) at glutamine 519, thereby inactivating its deacetylase activity as well as its ability to degrade viral proteins and activate interferon responses [94]. Lastly, nsp5-mediated nucleocapsid protein processing is speculated to be associated with PEDV adaptation to Vero E6 cells [72]. However, it is necessary to clarify the impact of nsp5 on the pathogenicity and virulence of PEDV in pigs (especially in piglets) before constructing recombinant PEDV LAV candidates via mutating the nsp5 gene using reverse genetic techniques.

#### 2.3.6. Nsp6

The nsp6 protein is the critical executor of PEDV-triggered autophagy in IPEC-J2 cells [95]. The exogenous expression of the nsp6 protein significantly reduces the levels of phosphorylated forms of autophagy suppressers mTOR and p70S6K and raises the level of the phosphorylated form of autophagy activator p53. At the same time, inhibiting AKT significantly decreases the level of phosphorylated mTOR and increases autophagy indicator LC3-II [95]. These results suggest that nsp6 induces PI3K/AKT/mTOR-mediated autophagy in IPEC-J2 cells. A proteomic study showed that PEDV nsp6 combined with the glucosyltransferase Rab-like GTPase activator and myotubularin domain containing 4 (GRAMD4), and promoted the apoptotic degradation of GRAMD4 via the PERK and IRE1 signaling pathways [96]. The N-terminal domain of nsp6 is essential for GRAMD4 degradation, while it does not affect its binding with GRAMD4.

#### 2.3.7. Nsp7

PEDV nsp7 also antagonizes the type I IFN signaling pathway by interfering with the activity of key proteins in an nsp8-independen manner [45,98,99]. Mechanistically, PEDV nsp7 specially interacts with the caspase activation and recruitment domains (CARDs) of melanoma differentiation-associated gene 5 (MDA5), interfering with the association of MDA5 with the protein phosphatase 1 (PP1). This interference suppresses type I IFN production via the MDA5-MAVS signaling pathway [98]. Meanwhile, the nsp7 protein blocks type I IFN signaling by interfering with the entry of key proteins into the nucleus [99]. PEDV nsp7 binds to the DNA binding domain of STAT1/STAT2, interfering with the association between Karyopherin subunit α1 (KPNA1) and STAT1/2, thereby impeding the entry of IFN-stimulated gene factor 3 (ISGF3) into the nucleus to start the transcription of ISGs.

#### 2.3.8. Nsp14

PEDV nsp14 is a bifunctional protein, including an N-terminal exoribonuclease activity domain (ExoN) and a C-terminal guanine-N7-methyltransferase activity domain (N7-MTase), which are critical for replication fidelity, mRNA stability, translation, and immune evasion [45,46,104,106]. PEDV nsp14 down-regulates the production of type I and type III IFN induced by poly (I:C) [45,46]. Furthermore, the overexpression of nsp14 inhibits the levels of PEDV- and SeV-induced IFN-β and MHC-I and promotes PEDV proliferation [102,106]. The N7-MTase activity of nsp14 plays an important role in regulating PEDV replication and antagonizing the IFN response [104]. Complete inactivation of N7-MTase activity in nsp14 is lethal to PEDV. However, the mutant rPEDV-D350A, generated using an RGS with a single mutation (D350A) in nsp14 retaining 29.0% of N7-MTase activity, is viable, but shows significant proliferation defects in Vero CCL-81 cells and intestinal porcine epithelial cells (IPEC-DQ). Interestingly, rPEDV-D350A remains genetically stable in Vero CCL-81 cells for at least 13 passages, and induces significantly higher levels of type I and III IFN in IPEC-DQ cells compared with the parental strain [104]. The PEDV mutant E191A, with a single amino acid mutation at the Mg^2+^-binding site in the ExoN domain of nsp14, replicates poorly in vitro and in vivo, and shows attenuation in neonatal piglets [105]. However, the E191A mutant is unstable both in vivo and in vitro, suggesting that the ExoN domain may not be a suitable target for PEDV LAV development. Additionally, PEDV nsp14 inhibits the SeV- or poly (I:C)-induced activation of the NF-κB signaling pathway and the early production of proinflammatory cytokines [106]. Nsp14 interacts with and inhibits the phosphorylation of IKKα/β and p65, thereby blocking the nuclear translocation of NF-κB [106]. Moreover, PEDV nsp14 inhibits the expression of ER stress-induced GRP78 in an N7-MTase-dependent manner [107]. Mechanistically, nsp14 represses the transcription and translation of GRP78 by inhibiting the activity of the GRP78 promoter and interfering with host translation, respectively.

#### 2.3.9. Nsp15

Nsp15, a conserved component of the coronavirus replication/transcription complex (RTC), exerts uridine-specific endoribonuclease (EndoU) activity [97]. Transcriptome analysis has shown that the overexpression of PEDV nsp15 in IPEC-J2 cells inhibits the expression of a number of genes involved in immune responses and inflammation, such as CCL5, CXCL8, CXCL10, OAS, MXs, STAT1, and IRF9. This suggests that nsp15 can antagonize INFs and block cytokine production to provide a beneficial intracellular environment for virus proliferation [112]. PEDV nsp15 EndoU activity is crucial for suppressing type I and type III interferon responses in macrophages and epithelial cells, thereby facilitating virus replication, shedding, and pathogenesis in vivo [108]. In comparison to the parental strain, the PEDV EndoU-H226A mutant induces an early and robust production of type I and type III IFNs as well as ISGs in porcine epithelial cells, leading to attenuation in piglets [108]. Mechanistically, nsp15 cleaves the 5′-polyuridine (polyU) from 5′-polyU-containing, negative-sense viral RNA, an intermediate product of virus replication acting as an MDA5-dependent pathogen-associated molecular pattern (PAMP), to evade innate immune responses [110]. Furthermore, PEDV nsp15 subverts type I and III IFN response and facilitates virus replication by directly degrading the RNA levels of TBK1 and IRF3 via its EndoU activity, in which residues of H226, H241, and K282 but not D265 are critical for this endoribonuclease activity [109]. In addition, PEDV nsp15 interferes with anti-viral stress granule (SG) formation to ensure efficient virus replication in an EndoU activity-dependent manner [111]. Collectively, these results indicate that nsp15 functions as an important virulence factor for PEDV and is a potential target for the development of antivirals and vaccines.

#### 2.3.10. Nsp16

PEDV nsp16 has 2′-O-methyltransferase (2′-O-MTase) activity, and serves as a potent antagonist of host innate immune responses. The expression of nsp16 not only antagonizes the production of type I and type III IFNs [45,46], but also inhibits the mRNA levels of innate immune-related genes induced by PEDV or SeV, such as IL-6, TNF-α, MHC-I, and MHC-II, to promote viral proliferation [102]. Mechanistically, nsp16 significantly inhibits the RIG-I- and MDA5-mediated IFN-β production and interferon-stimulated response element (ISRE) activation via its 2′-O-MTase activity, dependent on the catalytic tetrad K^45^D^129^K^169^E^202^ [102]. Studies on recombinant PEDV carrying mutant nsp16 generated using an RGS have confirmed the beneficial effect of nsp16 on virus replication in vitro and in vivo [113]. Hou et al. found that compared with the parental strain, PC22A, recombinant PEDVs with single (D129A) or quadruple alanine-substitutions (KDKE^4A^) in the catalytic tetrad of 2′-O-MTase were more sensitive to IFN-β, induced stronger type I and type III IFN responses, and replicated less efficiently in vitro. Compared with the parental strain and D129A mutant, the KDKE^4A^ mutant was significantly attenuated in Gn piglets [113].

#### 2.3.11. Other Nsps

Other nsps of PEDV also play a role in regulating the host’s innate immune responses to facilitate virus replication. For example, nsp8 has been shown to suppress type III IFN activities by reducing IRF1 promoter activity [46]. Nsp9 can up-regulate and interact with histone cluster 2 (H2BE), which inhibits PEDV-induced ER stress-mediated apoptosis, to facilitate PEDV replication [100]. In addition, PEDV nsp9 can also interact with heterogeneous nuclear ribonucleoprotein A3 (HNRNPA3), whose expression is down-regulated by PEDV infection in vitro and in vivo, leading to increased cellular lipid accumulation that provides a favorable environment for PEDV replication [101]. Although nsp10 alone is not an IFN antagonist, it can enhance the inhibitory effect of nsp16 on type I IFNs by activating the 2′-O-MTase activity of nsp16 [102]. The overexpression of nsp13 could significantly decrease the mRNA level of MHC-I in PEDV-infected cells, but it has no regulatory effect on other immune molecules, such as IFN-β, IL-6, and TNF-α [102]. In addition, nsp13 also down-regulates neonatal Fc receptor (FcRn) expression to facilitate the immune escape of PEDV by causing promoter hypermethylation in vitro and in vivo [103]. Nsp13 stimulates the overexpression of DNA methyltransferase 3b (DNMT3b) by activating the NF-κB signaling pathway, resulting in abnormal hypermethylation of the FcRn gene promoter region.

Although extensive research has been conducted on the functions of the structural and nonstructural proteins of PEDV, as well their potential impact on virus replication and virulence, most of these studies were performed under in vitro conditions of the overexpression of these proteins. The findings in these studies need to be validated by constructing recombinant PEDVs carrying one or more protein mutants, especially under in vivo conditions of recombinant virus infection, to verify the impact of the virulence factor mutants on the replication and pathogenicity of the recombinant virus, providing guidance for the generation of PEDV LAV candidates.

## 3. RGSs for PEDV

**Figure 2:**
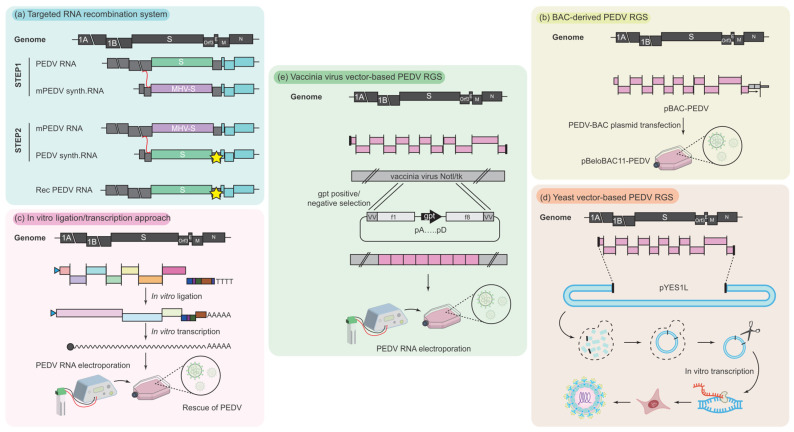
A diagrammatic representation of the development and design of next-generation live attenuated vaccines for PEDV using various reverse genetic systems (RGSs). (**a**) Targeted RNA recombination scheme for creating recombinant PEDV with ORF3 gene deletion/substitution. This scheme can be used to produce the interspecies chimeric virus mPEDV (Step 1) or recombinant PEDV variants lacking the ORF3 gene (Step 2). Synthetic RNAs transcribed from transfer vectors are introduced into PEDV- (Step 1) or mPEDV (Step 2)-infected cells through electroporation. A single recombination event within the 3′ region of ORF1b in the donor RNA and viral genome results in the generation of a recombinant genome. Selection of recombinant progeny viruses is based on their ability to form plaques in murine cell (LR7) monolayers (Step 1) or infect Vero cells while losing the ability to infect LR7 cells (Step 2). (**b**) BAC-derived PEDV reverse genetic system. The diagram displays the genome structure of PEDV and the genome fragments used for cloning the entire viral genome into a BAC plasmid (pBeloBAC11). The complete viral genome is divided into twelve fragments, with the CMV promoter at the 5′ end and HDVr and BGH sequences at the 3′ end. Transfection of the PEDV BAC plasmid into Vero cells leads to the rescue of PEDV. (**c**) Vaccinia virus vector-based reverse genetics system for PEDV. The PEDV genome is divided into multiple fragments, which are used to construct intermediate plasmids. These plasmids cover the entire PEDV genome and are introduced into the vaccinia virus genome through homologous recombination with GPT as a positive or negative selection marker. After linearization of the vaccinia virus genome by endonuclease digestion, the infectious mRNA is transcribed and electroporated into Vero cells to rescue recombinant PEDV. (**d**) An in vitro ligation/transcription approach for generating recombinant PEDV. The diagram shows the structure of the PEDV genome with the in vitro ligation method. The full-length cDNA of PEDV is assembled directionally using in vitro ligation, with a T7 promoter at the 5′ end and a poly (A) tail at the 3′ end. The assembled full-length cDNA is transcribed into genomic RNA in vitro and then introduced into Vero cells through electroporation to rescue recombinant PEDV. (**e**) Yeast vector-based PEDV reverse genetic system. The diagram shows the genome structure of PEDV and the overlapping DNA fragments used to clone the PEDV genome into a YAC vector (pYES1L). The first and last fragments have overlapping sequences for the YAC vector, with the CMV promoter fused to the 5′ end of the viral genome and HDVr and BGH added after the poly (A) sequence at the 3′ end. All cDNA fragments are transformed with a linearized YAC vector (pYES1L) into yeast competent cells for assembly through transformation-associated recombination. Positive clones are identified and extracted, and the full-length cDNA clones are transfected into Vero cells for virus recovery. In this figure, five reverse genetic systems (RGSs) utilized in the development of PEDV vaccines are depicted with distinct colored backgrounds (This schematic diagram is the author’s own creation).

The reverse genetic system (RGS) provides a powerful tool for studying virus characteristics and developing antivirals and vaccines from a holistic perspective. This includes understanding the expression regulation of viral genes, the function of viral proteins, the interaction between the virus and the host, and the rational design of LAVs. To date, several techniques, including targeted RNA recombination, in vitro ligation, bacterial artificial chromosome (BAC)-based ligation, vaccinia virus-based recombination, and transformation-associated recombination (TAR), have been employed to establish RGSs for PEDV (Figure 2). And these RGSs have been used to study the functions of several PEDV structural and nonstructural proteins and in generating PEDV LAV candidates [50,80,104,114,140,155]. Recently, Jiang et al. reviewed the principles, advantages, and drawbacks of the established RGSs for the emerging and re-emerging swine coronaviruses, including PEDV [156]. Thus, we will only provide a brief overview of PEDVs’ RGSs.

In cooperation with scientists from the Netherlands, we established the first RGS for PEDV (Figure 2a) [157]. Although this RNA-targeted recombination-based technique can only be used to introduce mutation or gene replacement into the last one-third of the PEDV genome, it is highly efficient in rescuing recombinant PEDV. It has been used to study the function of the S protein [155,158,159], intracellular transport and the function of ORF3 [30,140], and to develop PED LAV candidates (our unpublished data). In order to manipulate the whole genome of PEDV, bacterial artificial chromosome (BAC)-based ligation [160,161] and in vitro ligation [150,162] were subsequently employed to construct the full-length infectious cDNA clone of PEDV (Figure 2b,c). Currently, RGSs based on these two techniques are the most commonly used in PEDV research [50,80,104,152,163]. To avoid the tedious cloning process of generating the full-length cDNA of PEDV in vitro and take advantage of the transform-associated recombination (TAR) cloning system in yeast, Zhou et al. developed a TAR-based (also known as BAC/YAC-based) RGS for PEDV by using a hybrid vector containing a yeast artificial chromosome (YAC) and a BAC (Figure 2d) [164]. This one-step assembly of an infectious PEDV cDNA clone can be completed in one week with superior efficiency. Additionally, the CRISPR/Cas9 and homologous recombination techniques have been found to be simpler and faster than restriction enzyme digestion and ligation methods in editing PEDV cDNA clones, further improving the efficiency of using BAC- and YAC/BAC-based RGSs to generate recombinant PEDV [51,164,165,166]. Recently, Kristen-Burmann et al. developed a vaccinia virus-based RGS for PEDV through vaccinia virus-mediated homologous recombination (Figure 2e) [151]. This method allows for the stable propagation and efficient manipulation of full-length PEDV cDNA clones.

Two other RGS techniques based on infectious subgenomic amplicons (ISAs) [167] and circular polymerase extension reactions (CPERs) [168] have been used for genome manipulation and LAV development for SARS-CoV-2 and other positive-stranded RNA viruses, but have not yet been applied to PEDV research. Although these two methods claim to be swift and simple, it is necessary to verify their efficiency in rescuing recombinant viruses compared to the existing methods.

It must be pointed out that biosafety issues should be seriously considered when working with PEDV and developing PED LAVs using RGSs. To avoid virus transmission and environmental pollution, a series of biosafety guidelines and measures need to be followed, including using qualified laboratory facilities, strict operation procedures, sufficient personal protective equipment, and appropriate sample processing and waste disposal procedures. All the operations should be carried out in accordance with the prescribed procedures in the corresponding laws of the regions or countries where the work is carried out.

## 4. Developing PED LAV Candidates with RGSs

Given that the structural and nonstructural proteins of PEDV can antagonize the host’s innate immune responses at differential stages of viral replication and pathogenesis, facilitating viral replication in the intestine and leading to severe diarrhea or even death in neonatal piglets, theoretically, inactivating these viral virulence factors using RGSs holds the potential to reduce the virulence of resulting recombinant PEDVs. These attenuated strains could serve as PED LAV candidates. However, challenges such as the compromised host immune system and the genetic instability of attenuated strains due to recombination, revertant mutations, or complementary mutations elsewhere in the virus genome may contribute to the reversion of virulence of PED LAV strains. To address the risk of virulence reversion resulting from genetic instability, introducing multiple mutations into different genes and/or combining different attenuation strategies are usually employed to generate PED LAV candidates. In addition, as structural proteins, especially the S protein that determines the cell/host tropism of PEDV, play a crucial role in inducing humoral and cellular immunities, achieving a balance between the antigenicity, replication-capacity, and virulence/pathogenicity of recombinant PEDV is crucial for developing an ideal PED LAV candidate using an RGS. To date, several studies have attempted to modify the PEDV genome through RGS platforms, primarily focusing on improving the in vitro proliferation capabilities of recombinant PEDVs and reducing their virulence to the host by modifying the S gene or nsp genes. Table 2 summarizes the characteristics of PEDV LAV candidates generated using RGSs.

### 4.1. Constructing PED LAV Candidates by Modifying the S Gene

The PEDV S protein not only facilitates virus entry into host cells, but also serves as the main inducer of neutralizing antibodies in infected/immunized pigs. Therefore, as the key determinant of cell tropism, mutations in the S protein may alter its binding capability to host receptors, thereby affecting the proliferation characteristics of PEDV in vitro and in vivo, as well as its pathogenicity to the host [169]. Additionally, given its role in antibody induction, mutations in the S protein can lead to alterations in the antigenicity and immunogenicity of PEDV, thereby affecting the neutralization spectrum of the antibodies it elicits [170]. Some studies have used RGSs to modify the S gene of PEDVs with the aim of enhancing the proliferation capability of the recombinant viruses in Vero cells, which are commonly used for virus isolation and PED vaccine production. These modifications also seek to reduce the virulence of the recombinant viruses in vivo, ultimately generating potential candidates for PEDV LAVs. 

#### 4.1.1. Enabling PEDV Vero Cell Tropism by Modifying the S Gene

Virus isolation is crucial for studying the pathogenesis of the emerging and re-emerging viral pathogens and developing prophylactic vaccines. Although PEDV can infect cells of several species, the in vitro isolation and subsequent propagation of PEDV from clinical samples pose significant challenges [151,171]. As proliferation in cell lines derived from non-natural hosts is a kind of tropism switch for viruses, and the S protein of coronavirus is responsible for binding to the receptor and mediating the subsequent virus entry into the cell, several studies have tried to identify the key motifs on the S protein that determine the tropism of PEDV for Vero cells by using RGSs. Li et al. found that replacing the S1 and the first half of the S2 (S3) of the non-Vero cell-tropic CH/SX/2016 strain with the corresponding segments from the Vero cell-tropic HNXP strain allowed the resulting rCH/SX/2016- S1_HNXP_ + S3_HNXP_ to replicate efficiently in Vero cells. This suggests that the cooperation between S1 and S3 contributes to the Vero cell tropism of PEDV [172]. In particular, specific amino acid substitutions in the S2 subunit, such as 803L and 976H, were found to be crucial for the proliferation of rCH/SX/2016-S1_HNXP_ + S3_HNXP_ in Vero cells. Using PEDV DR13 as a model strain, we identified three key amino acid substitutions (A605E, E633Q, and R891G) in the S protein that enabled the attenuated PEDV strain DR13^att^ to efficiently infect and replicate in Vero cells [173]. We hypothesize that the three mutations change the viral tropism by changing the S2′ cleavage site and its RBD–receptor interaction. In a separate study, Kristen-Burmann et al. endowed the PEDV clinical strain MN with proliferation capability in Vero cells by introducing two amino acid mutations (L375F and H486P) to the S protein [151]. In addition, a tri-nucleotide insertion in the S gene (leading to D466GH mutation in S1) enabled two highly virulent PEDV US strains to induce syncytia and enhanced their proliferation capabilities in Vero cells [108,117]. However, the determinants of Vero cell tropism in PEDV found in these studies are all strain-specific, except for the D466GH mutation, which enhances Vero cell adaptability of two US strains. Moreover, these studies did not further validate the generalizability of their findings with other PEDV strains. Therefore, more studies are warranted to elucidate the determinants and the mechanism of Vero cell tropism in PEDV and to develop PED LAV candidates for timely responses to PED outbreaks caused by emerging PEDV variants.

#### 4.1.2. Enabling PEDV Trypsin-Independent Proliferation by Modifying the S Gene

In addition to the difficulties in isolating clinical strains, another constraint in PED LAV development is that the efficient proliferation of PEDV isolates in vitro usually requires the addition of exogenous proteases (mainly trypsin), which undoubtedly increases the complexity and cost of vaccine production. Elucidating the role of trypsin in the spatiotemporal process of PEDV infection and generating trypsin-independent PEDV using RGSs will facilitate PED LAV development. Several studies have focused on the role and the acting mechanism of trypsin in PEDV infection and showed that the S protein was the determinant of the trypsin dependence of both G1- and G2-type PEDVs. Trypsin mediates S protein cleavage and membrane fusion during trypsin-dependent PEDV entry [155,174,175].

Witch et. al. showed that trypsin cleaved the S protein of G1 PEDV CV777 after the virus bound to the receptor [155]. By swapping S gene fragments using an RGS, the determinant for trypsin-dependent cell entry was located at the N-terminal of the S2 subunit (890–1116 aa), including the fusion peptide and heptad repeat region 1 (HR1). Specifically, they speculate that the cleavage site (S2′, R890 in CV777) upstream of the fusion peptide was critical for S protein priming as swapping it with a furin cleavage site enabled the recombinant PEDV to grow efficiently without trypsin [158]. However, later studies have shown that the S2′ site is crucial for PEDV adaptation to cell lines, but not for trypsin dependence [54,155,163,173,174]. Another amino acid (Y976/977) adjacent to the HR1 domain of the S protein was speculated to be crucial for viral trypsin-independent phenotypes [54,175]. However, a subsequent study by Li et al. showed that Y976 on the S protein was also not the determinant of trypsin dependence in the trypsin-dependent PEDV AJ1102 [163]. Kim et al. identified six amino acid variations (M214I, A457T, A777P, Q825H, I958T, and Y976D) in the S protein by comparing the S protein sequence of the trypsin-independent PEDV 8aa P70 with that of its trypsin-dependent parental strain P0 [175]. Four of the six variations were located near the S1/S2 junction and fusion peptide. The mutations responsible for the trypsin independence of P70 need further investigation through the construction of recombinant PEDVs using RGSs. Lately, two research groups attempted to identify the trypsin dependency determinant of G2-type PEDV using BAC-based full-length infectious clones, and found that the trypsin dependency determinant resides in the S2 subunit rather than directly at the trypsin cleavage site of the S protein [163,174]. Tan et al. located the trypsin dependency determinant of PEDV YN200 in the S2 (727–1383 aa) subunit, rather than the S1 (1–726 aa) or the N-terminal fragment of the S2 subunit (NS2′, 727–892 aa), suggesting the vital role of the S2 subunit in PEDVs’ trypsin dependence [174]. Li et al. further narrowed down the trypsin dependency determinant to the S2′ (894–1386 aa) subdomain of the trypsin-dependent G2 PEDV AJ1102 S protein [163]. However, although the N-terminal half (including the fusion peptide and fragment ahead of HR1, 894–993 aa) and the C-terminal half (from HR1 to the end of the S protein, 994–1386 aa) of the S2 subunit have been shown not to be the trypsin dependency determinant of PEDV AJ1102 [163], further experiments are warranted to verify whether the N-terminal fragment of the S2 subunit containing the putative S2′ cleavage site, fusion peptide, and HR1 domain (i.e., 894–1140 aa) is the trypsin dependency determinant of rAJ1102, as discovered in PEDV CV777 by Witch at el. [155].

Interestingly, once PEDV becomes trypsin-independent, its replication is severely impaired by trypsin, which inhibits virus attachment and entry into cells [155,163]. Although the pre-treatment of Vero cells with trypsin does not affect the replication of trypsin-independent PEDV, the pre-treatment of trypsin-independent PEDV with trypsin significantly reduces virus attachment and titer [54,155,175]. Incubation-purified trypsin-dependent PEDV or its S protein with trypsin leads to only partial cleavage of the S protein, but trypsin cleaves essentially all the S protein of trypsin-independent PEDV, resulting in two fragments [155] or just a smear lane on gel [174]. Both studies indicate that the S protein of trypsin-dependent PEDVs is relatively resistant to trypsin before binding to the receptor, while the S protein of trypsin-independent PEDVs is readily cleaved or degraded by trypsin even before binding to the receptor. The pre-priming or degradation of the S protein of trypsin-independent PEDV by trypsin before binding to the receptor may impair the receptor binding and membrane fusion function of the S protein, thus leading to the observed significant decrease in infectivity of the trypsin-independent PEDV in the presence of trypsin. Kim et. al. investigated the effect of trypsin on the proliferation of trypsin-independent PEDV 8aa in Vero cells using a fluorescence confocal microscope. They found that trypsin inhibited the escape of PEDV 8aa from endosomes, while the virus rapidly left the endosomal compartments and initiated replication in the cytoplasm in the absence of trypsin, suggesting that trypsin impaired the trafficking of the trypsin-independent PEDV during the entry stage [175].

It is worth noting that these studies are based on the analysis of different PEDV strains, which may result in discrepancies in the determinants of PEDV trypsin independence. In addition, although immunization with rAJ1102-S2′_JS2008_ induced neutralizing antibodies against both AJ1102 and JS2008 [163], substitution with S2′_JS2008_ might impair the protective efficiency of rAJ1102-S2′_JS2008_ against epidemic G2 PEDVs. Therefore, in order to construct a trypsin-independent recombinant PEDV via RGSs as a PED LAV candidate to promptly respond to the emerging PED epidemic, it is necessary to identify the conserved key residue(s) on the S protein responsible for the trypsin-independent proliferation of various epidemic G2 PEDV strains by generating isogenic recombinant viruses with the same background genes.

#### 4.1.3. Attenuating PEDV by Modifying the S Gene

Due to the fact that the S protein is not only the determinant of PEDV tropism and pathogenicity, but also the main inducer of neutralizing antibodies, it is an ideal modification target for constructing PED LAV candidates using RGSs. As variations in the S protein of PEDV variant strains (G2) are the main contributors resulting in the inability of vaccines based on PEDV classical strains (G1) to provide sufficient protection against G2 PEDV strains, researchers first attempted to generate a PED LAV candidate via replacing the intact or partial S gene of an attenuated G1 strain with the corresponding segment of a clinical G2 strain by using an RGS in order to promptly control the PED epidemic caused by G2 strains. However, replacing the entire S gene from a highly virulent strain can sometimes make the initially attenuated PEDV pathogenic to piglets [49,114,115]. Although substituting the S gene with that of a highly virulent G2 strain (BJ2011C) did not result in virulence reversion of an avirulent G1 strain (CHM2013) [114], substitution with the entire S gene from virulent G2 PEDV resulted in the virulence reversion of the avirulent PEDV PT-P96 (G2) [115] and CV7777 (G1) [49]. We generated a recombinant PEDV by incorporating the extracellular domain coding part of the S gene from an epidemic strain into an avirulent G1 PEDV DR13-like strain. Our unpublished data indicate that the recombinant strain was avirulent in neonatal piglets. Based on the fact that PEDV clinical isolates with a large fragment deletion (582–648 nucleotides) in the 5′-terminal region of the S gene show reduced virulence in piglets [116,176], researchers have also attempted to attenuate PEDV by replacing or deleting a partial S gene, with some success. Unlike the recombinant PEDV CV777 carrying the S1 gene from a highly virulent Japanese strain, which regained high pathogenicity to piglets, resulting in severe watery diarrhea, dehydration, and 100% mortality in infected Gn piglets [49], PEDV icPC22A-S1Δ197, which bore a 197-aa deletion in the N-terminal of the S1 subunit, was attenuated, inducing only mild-to-moderate diarrhea and no mortality in neonatal Gn piglets (Table 2) [117]. Although vaccination/challenge experiments with the recombinant strain icPC22A-S1Δ197 were not conducted, an infection/challenge experiment with another strain, TC-PC177, which had the same deletion in the S protein, indicated that primary infection with TC-PC1777 could provide partial cross-protection for suckling piglets against highly virulent PEDV [117]. 

The PEDV S protein contains two intracellular sorting motifs, YxxΦ (a tyrosine-based endocytosis signal, YEVF or YEAF) and KVHVQ (an endoplasmic reticulum retrieval signal) at the cytoplasmic tail. The deletion of the C-terminal tail has been observed in clinical isolates or cell passaged attenuated PEDVs [51,161]. The deletion or inactivation of the conserved motifs (YxxΦ and KVHVQ) by using RGSs can reduce the viral pathogenicity to piglets (Table 2) [50,51]. This deletion can also result in higher levels of IgG, IgA, and neutralizing antibodies, and enhances protection effects against virus challenges [51]. As no neutralizing epitopes are located in this region, the deletion of these two motifs not only increases the proportion of S proteins transported to the surface of virus-infected cells for recognition by the host’s immune system, but also does not reduce their immunogenicity. Therefore, in the future, the inactivation/deletion of the two motifs in the S protein is worth more attention in PEDV LAV development. Furthermore, additional experiments are needed to validate the efficacies of these LAV candidates in pregnant sows to investigate their ability to induce sufficient antibodies, especially sIgA, in colostrum and milk. This is essential for protecting their offspring against highly virulent PEDV infections.

### 4.2. Constructing PED LAV Candidates by Modifying Nsps

Compared to G1 PEDV, G2 PEDV is more resistant to IFNs and replication restriction by complement C3 [81,177]. Inactivating viral interferon antagonists using RGSs could reduce the virulence of various viruses [80]. Studies have shown that mutations in one or several structural/nonstructural proteins can attenuate PEDV to a certain extent (Table 2). However, to become an ideal PED LAV candidate, the mutant must be safe for neonatal piglets and capable of equipping neonatal piglets with effective passive anti-PEDV immunity in the form of colostrum/milk-derived antibodies via the immunization of pregnant sows.

Nsp1, nsp2, and nsp14–16 have been targeted for mutation using RGSs to attenuate G2 PEDVs (Table 2). As the most potent antagonist of host innate immune responses, the mutation of PEDV nsp1 can attenuate viral virulence. For example, Niu et al. generated recombinant PEDV N93/95A by mutating nps1 N93 and N95, which are crucial for their IFN-antagonizing functions, to A93 and A95 using an RGS with the cDNA of a highly virulent PEDV PC22A as a backbone [153]. A challenging experiment in Gn neonatal piglets showed that mutant N93/95A was partially attenuated but retained viral immunogenicity (Table 2) and remained genetically stable in vitro and in vivo. In another study by Deng et al., an F44A mutation in nsp1 was incorporated into the PEDV Colorado strain to generate an Nsp1mt mutant using an RGS [80]. Although the Nsp1mt mutant replicated less efficiently in IFN-competent PK1 cells, its pathogenicity was not evaluated in this study. Recently, Fan et. al. constructed a PEDV mutant, r12/NSP1 (V50A), by mutating nsp1 V^50^, the key amino acid in regulating C3 expression, to A^50^ using an RGS with virulent strain AH2012/12 as the backbone [81]. r12/NSP1 (V50A) lost the ability to suppress the expression of complement component 3 (C3), which inhibits viral replication. Similarly, the pathogenicity of this mutant has not been tested yet. Jiao et al. studied the role of nsp2 on the pathogenesis of PEDV by constructing a mutant without the entire nsp2 gene (rPEDV-Δnsp2) from a highly virulent strain using an RGS [83]. Although challenging studies have shown that rPEDV-Δnsp2 was avirulent in neonatal piglets (Table 2), inducing the robust expression of IFNs and ISGs, further studies on the immunization of pregnant sows with rPEDV-Δnsp2 are warranted to verify its capability to induce sufficient maternal immunity in the form of colostrum and milk in order to protect neonatal piglets against virulent PEDV. Both the ExoN and N7-MTase activity domains of Nsp14 were targeted for mutation using RGSs to study their effects on PEDV pathogenesis. Compared to its parental strain, rPEDV-D350A deficient in the N7-Mtase of nsp14 replicates poorly in both Vero CCL-81 and IPEC-DQ cells, inducing more robust production of type I and III IFNs in IPEC-DQ cells [104]. However, to use rPEDV-D350A as an LAV candidate for PED, it is necessary to verify its pathogenesis in neonatal piglets in advance. Niu et al. investigated the effect of nsp14-ExoN deficiency on PEDV pathogenesis [105]. They generated recombinant PEDV E191A by incorporating E^191^A in the Mg^2+^-binding site of the ExoN of PEDV PC22A using an RGS. The E191A mutant was non-lethal to 5-day-old Gn piglets, providing inoculated piglets with 100% mortality protection against virulent PEDV. However, the mutant was genetically instable in vitro and in vivo, suggesting nsp14-ExoN is a suitable target for drugs instead of vaccines. Deng et al. generated an icPEDV-EnUmt mutant by incorporating an H^226^ to A mutation to the EndoU catalytic site of PEDV Colorado nsp15 using an RGS [108]. Although the EnUmt mutant was non-lethal to neonatal piglets (Table 2), the intestinal lesions it induced in piglets suggested more mutations in nsp15 or other sites are needed to fully attenuate the lethal PEDV Colorado. Hou et al. tried to attenuate PEDV PC22A by inactivating the 2′-O-MTase activity of PEDV PC22A nsp16 [113]. A KDKE^4A^ mutant was generated using an RGS by mutating the catalytic tetrad K^45^-D^129^-K^169^-E^202^ of 2′-O-MTase to alanines. The KDKE^4A^ mutant is significantly attenuated in neonatal Gn piglets, and inoculating 4-day-old piglets with KDKE^4A^ induced an 80% protection from diarrhea following a challenge with virulent icPC22A (Table 2).

To circumvent the reversion of the recombinant virus to a virulent phenotype and fully attenuate virulent PEDVs, researchers also tried to use RGSs to introduce mutations into multiple virulence-related genes in order to construct PEDV LAV candidates. KDKE^4A^-SYA obtained by further inactivating the endocytosis signal of the spike protein of the KDKE^4A^ strain was fully attenuated in neonatal Gn piglets, showing minimal clinical signs and no death. KDKE^4A^-SYA provided sufficient (100%) protection from diarrhea following a high dose challenge with the highly virulent parental PEDV icPC22A. Furthermore, KDKE^4A^-SYA retained the introduced mutations after three passages in pigs, suggesting it is genetically stable in vivo [113]. In a study by Deng et al., the recombinant strain icPEDV-mut4, which had inactive versions of three viral IFN antagonists (nsp1, nsp15, and nsp16), was shown to be highly attenuated. It replicated in the intestine of piglets without causing diarrhea but was able to induce specific IgG and neutralizing antibodies in virus-infected piglets (Table 2) [80]. Similarly, the introduced mutations were maintained in the viral RNA shed from the infected animals [80]. However, further experiments are warranted with sows to verify whether these recombinant PEDVs can elicit sufficient antibodies, especially sIgA, in colostrum and milk to provide protection for neonatal piglets from virulent PEDV infection. Additionally, it is also necessary to evaluate the genetic stability of mutants in adult pigs, which have a more mature immune system and can exert stronger selective pressure on the introduced mutations [113].

### 4.3. Constructing PED LAV Candidates with Other Genetic Modifications

In addition to the aforementioned nonstructural protein genes and the S gene, the E and ORF3 genes were also targeted to attenuate PEDV. Furthermore, other strategies addressing the recombination-driven virulence reversion for other coronavirus, including SARS-CoV-2, such as reconstructing transcriptional regulatory circuits and genomic recoding, have also been used to construct PED LAV candidates.

Based on the fact that ORF3 is dispensable for the proliferation of PEDV both in vitro and in vivo [150,157] and the absence of ORF3 has little effect on the virulence and antigenicity of the recombinant virus in piglets [150], it can be replaced by other antigen genes of another porcine pathogen to generate divalent vaccines. Li et al. tried to construct a live PEDV-rotavirus divalent vaccine by swapping the ORF3 gene of an attenuated PEDV YN150 with the rotavirus VP7 gene using an RGS [178]. The chimeric virus displayed similar phenotypes both in vitro and in vivo, and induced high levels of specific neutralizing antibodies against both viruses simultaneously in vaccinated piglets. Further sow inoculation experiments and challenge experiments using highly virulent PEDV and rotavirus strains are warranted to verify the protective efficacy of the bivalent vaccine candidate on neonatal piglets. 

As a transmembrane structural protein of coronaviruses with ion channel activity, the E protein affects viral morphogenesis and virulence, but with significant differences among coronaviruses due to unknown reasons [179]. For instance, the E gene is absolutely essential for the production of infection with TGEV and Middle East respiratory syndrome coronavirus (MERS-CoV); the E gene is dispensable for the replication of murine hepatitis virus (MHV) and severe acute respiratory syndrome coronavirus-1 (SARS-CoV-1), and ΔE usually leads to aberrant morphology of the recombinant virus, reduces the number of mature virions, and attenuates the virus. A 7-aa deletion (23–29 aa) in the E protein was found in several G1 PEDVs, as well as in the attenuated DR13 strain, but not in the virulent counterpart [57]. To test the effects of the 7-aa deletion on PEDV virulence, Li et al. constructed a recombinant PEDV-E_Δaa23–aa29_ and showed that deletion of the 7-aa fragment in the E protein attenuated PEDV while retaining its immunogenicity (Table 2), and the mutant was genetically stable in vitro and in vivo [57]. Compared with the parental strain, PEDV-E_Δaa23–aa29_ caused lower mortality (33%) in piglets and provided protection for the surviving piglets against the challenge with virulent PEDV. We tried to generate a recombinant PEDV-ΔE but failed (our unpublished data), suggesting that the E gene might be essential for PEDV propagation as found for TGEV. As the E protein plays multiple roles in coronavirus proliferation, more studies are warranted to elucidate the role of the E gene in PEDV replication, assembly, release, and pathogenicity, and ultimately generate PED LAV candidates based on these findings.

As recombinant PEDV variants, especially those generated by the recombination of an attenuated vaccine strain and a virulent field strain, have been detected in clinical samples from different areas [42,43,44,180,181], the recombination-driven reversion of virulence has raised significant concerns regarding the use of PED LAVs. One strategy to construct recombination-resistant PEDVs involves remodeling the transcriptional regulatory sequences–core sequences (TRS-CSs) of the genome, whose compatibility is critical for coronavirus RNA synthesis [13,182,183]. Wang’s group firstly tried to redesign the TRS-CSs of PEDV PC22A, and a remodeled TRS (RMT) mutant was constructed by replacing the TRS circuits with 5′-GUGAAU-3′ using an RGS [11]. The RMT mutant exhibited a phenotype comparable to its parental strain and was genetically stable in vitro and in vivo (Table 2). The co-infection assays of Vero cells and neonatal Gn piglets with the RMT mutant and another PEDV strain have shown that the RMT mutant was recombination-resistant both in vitro and in vivo. This RMT mutant can be further tuned, such as changing the length of remodeled TRS circuits, coupled with other attenuation strategies, to generate reversion-proof PED LAV candidates.

An alternative means to improve the genetic stability of PED LAVs is to recode the virus genome through designing the nucleotide sequence de novo without changing the amino acid sequences of viral proteins with the help of bioinformatics techniques. This strategy is also coined as synthetic attenuated virus engineering (SAVE), and has been applied in the development of LAVs for several viral diseases in humans and animals, including COVID-19 and porcine reproductive and respiratory syndrome (PRRS) [184,185,186,187]. This technology generates LAV candidates by genetically modifying the viral genome via (de)optimizing codons or codon pairs, increasing the content of CpG or UpA, or substituting serine and leucine codons with synonymous codons that could result in nonsense codons with a single-nucleotide mutation in one or more genes, thus reducing viral fitness while keeping their antigenicity [188]. At the same time, introducing hundreds or even thousands of mutations across the recoded genome significantly diminishes the likelihood of reverse mutations and homologous recombination. In one study, recombinant PEDV with a recoded N gene was generated [132]. An increase in the CpG content in the N gene renders the recoded virus more susceptible to ZAP and grows poorly in Vero cells [132]. Although this study did not continue with the animal immunization experiment to verify the virulence and immunogenicity of the recoded PEDV, the results emanated the potential of the SAVE technique in designing and developing PED LAV candidates. However, further efforts are necessary to recode additional structural/nonstructural proteins to generate ideal PED LAV candidates.

## 5. Future Perspectives in PED Vaccine Development

At present, the emerging and re-emerging PED epidemics are still threatening the healthy development of the global pig industry, damaging the safe supply of pork. Therefore, it is urgent to strengthen the cutting-edge basic research on the interaction between PEDV and hosts, ultimately leading to lethal diarrhea in piglets. The findings of these studies will lay the foundation for formulating intervention strategies and developing rationally designed LAVs for PED. The RGS enables the manipulation of the PEDV genome for multiple purposes, ranging from the identification of virulence factors to the rational design of LAV candidates. An ideal PED LAV candidate needs to achieve the optimal balance between safety and immunogenicity. This means that the vaccine candidate can induce a sufficient immune response in pregnant sows to provide neonates with adequate protection against infection by a highly virulent strain via milk or colostrum, without causing disease in neonatal piglets. Moreover, the vaccine strain should be genetically stable in vivo, especially in immunocompromised animals, such as sows with immunosuppressive diseases (e.g., African swine fever, PRRS, porcine circovirus disease, etc.) or newborn piglets with underdeveloped immune systems.

To achieve these goals, several unanswered questions should be focused on in the future: (1) The pathogenic mechanism of PEDV, especially the molecular basis leading to differences in pathogenicity and immune response induced by virulent and attenuated strains. The identification of key viral and host factors related to virulence will provide a theoretical basis for the rational design of attenuated LAV candidates and virus resistance breeding, respectively. (2) The quantitative relationship between the titers of neutralizing antibodies and the protection of neonatal piglets. Determining the minimum neutralizing antibody titer (IgG/IgA) required to protect piglets from highly virulent epidemic PEDV strains, preferably in sera that can be measured in advance before delivery, will provide a unified and reliable basis for the evaluation of vaccines and their clinical immune efficacy. (3) Optimal immunization strategies for sows. It is necessary to study the impact of multiple factors that might affect the gut–mammary gland–sIgA axis of sows on the efficacy of vaccines under clinical situations, including sow parity, gestation period, previous pathogen exposure, micronutrient supplementation (such as probiotics and vitamin A), etc. In addition, using appropriate mucosal adjuvants/delivery carriers (especially sustained-release adjuvants suitable for oral administration) and immunizing sows at the most sensitive intestinal lymphocyte stage will result in a more effective mucosal immune response in sows. (4) The genetic stability and safety of LAVs. To ensure the clinical safety of LAVs, multiple strategies, such as mutations in multiple genes, TRS rewiring, and genome recoding, should be combined to generate PED LAV candidates to reduce the virulence reversion of vaccine strains caused by mutations or recombination with clinical strains. (5) Antibody-dependent enhancement (ADE). Although the molecular mechanism awaits investigation, ADE found in other coronaviruses was also confirmed for PEDV [189,190]. One study identified an enhancing monoclonal antibody (mAbG3) against PEDV and localized its recognized epitope motif to the S1 subunit of the S protein [190]. Identifying and inactivating these enhancing epitopes in PED LAV candidates will benefit vaccine efficacy.

Porcine viral diarrhea, caused by co-infections and secondary infections by PEDV and other porcine enteric viruses, such as TGEV, porcine deltacoronavirus (PDCoV), and porcine rotavirus (PoRV), is very common on swine farms. A live recombinant viral vector vaccine presents an attractive approach to prevent the co-infection of porcine enteroviruses. Recently, Li et al. generated a PEDV and PoRV bivalent vaccine (rPEDV-PRoV-VP7) using PEDV YN150 as a backbone [178]. rPEDV-PRoV-VP7 exhibited excellent immunogenicity in piglets, inducing specific neutralizing antibodies against both PoRV and PEDV. This suggests that PEDV can serve as a live virus vector for expressing the antigen protein of another pathogen to develop a bivalent vaccine. Further research is warranted to develop bivalent or multivalent vaccines using PEDV as a vector for the prophylaxis of co-infection by porcine enteric pathogens.

Compared with intramuscular means, intradermal administration is more animal-friendly, and the antigen administrated can more readily access the dendritic cells in the dermis and the skin-draining lymph nodes. Thus, intradermal vaccination will enable the antigen in the vaccine to be more efficiently recognized by the immune system, inducing a more rapid immune response. Intradermal administration studies of vaccines for several animal and human diseases have shown that the efficacy of intradermal vaccination is better than that by intramuscular means [191,192,193,194,195]. Recently, Choe et al. intradermally vaccinated pregnant sows with a killed PED vaccine and showed that the intradermal vaccination could provide piglets with 70% mortality protection against virulent PEDV [196]. Although there is a lack of comparison with other delivery means in terms of protection rates, this study demonstrates the effectiveness of administrating the PED vaccine through the intradermal route. As a promising means to improve vaccine efficacy and spare vaccine doses, the intradermal delivery of PED LAVs deserves special attention, especially from comparative studies of the intradermal and oral administration of a commercial PED LAV.

Since the emergence of porcine respiratory coronavirus (PRCoV), a mutant of TGEV with altered respiratory tissue tropism and causing no or mild clinical signs, the prevalence of TGEV has decreased in PRCoV-seropositive herds due to cross-protective immunity with TGEV [197,198]. Recently, novel respiratory variants of porcine hemagglutinating encephalomyelitis virus (rvPHEV), exhibiting exclusively respiratory symptoms without the neurological symptoms typically associated with classical PHEV infection, have been identified in both China and the USA [199,200]. Although the mechanisms underlying the emergence of PRCoV and rvPHEV are still unclear, one cannot help but wonder if it is possible to generate an avirulent respiratory variant PEDV (rvPEDV) that only infects the respiratory tract. This may present one of the most attractive research directions for developing PED LAVs using RGSs in the future.

## 6. Conclusions

Despite the immunization of sows with inactivated or LAVs being common on pig farms, especially in Asian countries, PED epidemics caused by the emerging PEDV variants still cause substantial damage to the global pig industry. Hence, there is an urgent need for next-generation vaccines in clinical practice, preferably LAVs for PED. Although significant progress has been made in understanding the interactions between viral structural/nonstructural proteins and host cells, the pathogenic mechanism of PEDV remains unclear, especially the mechanism resulting in high mortality rates in piglets. RGSs provide a powerful platform for the rational modification of the PEDV genome to generate recombinant viruses, for studying the pathogen mechanism of PEDV, and for developing LAVs for PED. However, in order to design an ideal LAV for PED, further research is warranted to elucidate the questions mentioned above, such as the relationship between virulence and the activation of the gut–mammary gland–sIgA axis to produce high titer neutralizing antibodies in milk and the minimum immune dose for sows to protect piglets against heterogenous strains in clinical practice. In addition, the RGS approaches used to generate LAV candidates for other coronaviruses, especially SARS-CoV-2, which attracts the most attention [201,202], should also be considered in the development of PED LAV candidates using RGSs in the future.

Due to the fact that PEDV is the main pathogen of porcine viral diarrhea, developing a multivalent LAV using the PEDV genome as a backbone to cope with the co-infection-induced porcine viral diarrhea deserves special attention, and these vaccines will be more favored in clinical practice. As a novel vaccine administration means, whether the intradermal delivery of a PED LAV can trigger the mammary gland–sIgA response is an urgent question to be answered. If yes, this animal-friendly immune approach will effectively overcome the challenge of poor efficacy of PED LAVs delivered orally. Lastly, generating an avirulent rvPEDV using an RGS deserves to be tried, and it may be one of the most attractive measures for effectively preventing and controlling PED epidemics in the future.

## Figures and Tables

**Table 1:** The functions of PEDV virulent proteins.

Proteins	Biological Functions	Key Amino Acids	Reference
S (S1/S2)	(1) Receptor binding and virus entry;	/	[48]
	(2) A vital virulence factor;	^1377^YEVF^1380^; ^1382^KVHVQ^1386^	[49,50,51]
	(3) Inhibits the activity of the sodium–hydrogen ion transporter, Na^+^/H^+^ exchanger (NHE3);	/	[52]
	(4) Inhibits type I interferon (IFN) signaling;	/	[53]
	(5) Induces cell apoptosis.	/	[47]
E	(1) Virus assembly and budding;	/	[54]
	(2) Induces endoplasmic reticulum (ER) stress;	^16^LWLFV^20^ and L^25^	[54,55]
	(3) Activates nuclear factor-κB (NF-κB);	/	[55]
	(4) Up-regulates proinflammatory cytokine production;	^16^LWLFV^20^ and L^25^	[54,55]
	(5) Inhibits apoptosis;	^16^LWLFV^20^ and L^25^	[54,55]
	(6) Inhibits type I and III IFN production;	^23^FLLIISI^29^	[45,46,56,57,58]
	(7) Inhibits swine leukocyte antigen II DR (SLA-DR) expression.	/	[59]
M	(1) Virus assembly and morphogenesis;	/	[60,61]
	(2) Induces cell cycle arrest at the S phase;	/	[61]
	(3) Inhibits type I and III IFN production;	/	[45,46,60]
	(4) Interacts with heat shock protein 70 (HSP70).	/	[62]
N	(1) Virus replication and assembly;	/	[12]
	(2) Induces cell cycle arrest at the S phase;	^183^GRG^185^	[63,64,65]
	(3) Induces ER stress;	/	[63]
	(4) Up-regulates proinflammatory cytokine production;	/	[63]
	(5) Activates NF-κB;	/	[26]
	(6) Promotes p53 degradation;	/	[66]
	(7) Inhibits type I and III IFN production;	/	[45,67,68,69]
	(8) Increases the intracellular content of viral RNA;	/	[70]
	(9) Decreases Na^+^/H^+^ exchanger 3 (NHE3) on the cell membrane;	/	[71]
	(10) Cell culture adaptability.	/	[72]
ORF3	(1) Ion channel activity;	/	[73]
	(2) Inhibits apoptosis;	/	[30]
	(3) Induces autophagy;	/	[74]
	(4) Induces ER stress;	/	[74]
	(5) Inhibits type I and III IFN production;	/	[45,46]
	(6) Induces cell cycle arrest at the S phase;	/	[75]
	(7) Inhibits proinflammatory cytokine production;	/	[76]
	(8) Suppresses NF-κB activation;	/	[77]
	(9) Inhibits SLA-DR expression.	/	[59]
Nsp1	(1) Vital virulence factor;	/	[45,46,78]
	(2) Induces cell cycle arrest at the G0/G1 phase;	/	[79]
	(3) Suppresses type I and III IFN production and signaling;	N^93^/N^95^; F^44^	[45,46,80]
	(4) Inhibits NF-κB activation;	/	[78]
	(5) Reduces complement component 3 (C3) expression;	V^50^	[81]
	(6) Decreases peroxisome abundance;	/	[46]
	(7) Inhibits proinflammatory cytokine production;	/	[78]
	(8) Triggers host translation shutoff.	^92^SNCNY^96^	[82]
Nsp2	(1) Vital virulence factor;	/	[83]
	(2) Inhibits type I IFN production and signaling;	/	[83]
	(3) Targets F-box and WD repeat domain-containing 7 (FBXW7) for degradation;	/	[84]
	(4) Induces autophagy;		[83]
	(5) Recruits NBR1 for autophagic targeting of TANK binding kinase 1 (TBK1).	/	[83]
Nsp3	(1) Suppresses type I and III IFN production;	/	[45,46,85]
	(2) Deubiquitinates RIG-I and stimulator of interferon genes (STING);	C^1729^/H^1888^/D^1901^	[85]
	(3) Interacts with poly(C) binding protein 2 (PCBP2).	/	[86]
Nsp4	(1) Up-regulates proinflammatory cytokine production.	/	[87]
Nsp5	(1) Induces ER stress;	/	[88]
	(2) Induces protein kinase B (PKB/AKT) phosphorylation;	/	[23]
	(3) Promotes apoptosis;	H^41^/C^144^	[89]
	(4) Antagonizes pyroptosis;	/	[90]
	(5) Inhibits type I and III IFN production;	/	[46,91,92]
	(6) Cleaves key effectors of the RIG-I signaling pathway;	/	[91,92]
	(7) Cleaves porcine DNA polymerase delta interacting protein 3 (POLDIP3);	/	[93]
	(8) Induces the cleavage of lysophospholipid acyltransferases (LPACT3);	/	[88]
	(9) Cleaves histone deacetylase 6 (HDAC6).	/	[94]
Nsp6	(1) Induces autophagy;	/	[95]
	(2) Promotes ER stress;	/	[96]
	(3) Promotes apoptosis.	/	[96]
Nsp7	(1) Localizes within the replication/transcription complex (RTC);	/	[97]
	(2) Inhibits type I IFN production and signaling;	/	[45,98,99]
	(3) Inhibits NF-κB activation.	/	[98]
Nsp8	(1) Localizes within the RTC;	/	[97]
	(2) Inhibits type III IFN production.	/	[46]
Nsp9	(1) Localizes within the RTC;	/	[97]
	(2) Up-regulates and interacts with histone cluster 2 (H2BE);	/	[100]
	(3) Interacts with heterogeneous nuclear ribonucleoprotein A3 (HNRNPA3).	/	[101]
Nsp10	(1) Localizes within the RTC;	/	[97]
	(2) Enhances the inhibitory effect of nsp16 on type I IFNs.	/	[102]
Nsp12	(1) Localizes within the RTC.	/	[97]
Nsp13	(1) Localizes within the RTC;	/	[97]
	(2) Inhibits major histocompatibility complex I (MHC-I) expression;	/	[102]
	(3) Down-regulates neonatal Fc receptor (FcRn) expression;	/	[103]
	(4) Activates NF-κB.	/	[103]
Nsp14	(1) Localizes within the RTC;	/	[97]
	(2) Vital virulence factor;	D^350^, E^191^	[104,105]
	(3) Inhibits type I and III IFN production;	D^350^	[45,46,102,104,106]
	(4) Inhibits NF-κB activation;	/	[106]
	(5) Inhibits proinflammatory cytokine production;	/	[106]
	(6) Inhibits MHC-II expression;	/	[102]
	(7) Suppresses 78-kD glucose-regulated protein (GRP78) expression.	/	[107]
Nsp15	(1) Localizes within the RTC;	/	[97]
	(2) Vital virulence factor;	H^226^	[108]
	(3) Inhibits type I and III IFN production;	H^226^, H^241^, K^282^	[45,46,108,109]
	(4) Cleaves the 5′-polyuridines from negative-sense viral RNA;	/	[110]
	(5) Inhibits stress granule (SG) formation;	/	[111]
	(6) Down-regulates proinflammatory cytokine production.	/	[112]
Nsp16	(1) Localizes within the RTC;	/	[97]
	(2) Vital virulence factor;	K^45^D^129^K^169^E^202^	[113]
	(3) Inhibits type I and III IFN production;	K^45^D^129^K^169^E^202^	[45,46,102,113]
	(4) Inhibits MHC-I and MHC-II expression;	/	[102]
	(5) Down-regulates inflammatory cytokine production.	/	[102]

Note: / means not suitable or unknown.

**Table 2:** PED LAV candidates generated using RGSs.

Candidates	Modifications	Pathogenicity	Efficacy ^1^		Reference
			Vaccination	Challenge	
icPC22A-S1Δ197	Δ197 (34–230 aa) in S1	Gnotobiotic (Gn) piglets (5 d old, *n* = 4); 100 PFU/pig. (1) No mortality; (2) Fecal shedding↓; (3) Fecal score↓; (4) Viral antigens in epithelial cells↓; (5) Intestinal villus height to crypt depth (VH/CD) ratios↑.	/	/	[117]
icΔ10aa	Δ^1377^YEVFEKVHVQ^1386^ in S	Gn piglets (5 d old, *n* = 4); 100 PFU/pig. (1) Severe diarrhea rate↓; (2) Fecal shedding↓; (3) Intestinal VH/CD ratios↑.	/	/	[50]
rAH2012/12-Δ2	Δ^1380^FEKVHVQ^1386^ in S	Piglets (2 d old, *n* = 6); 2 × 10^5^ TCID_50_/pig. (1) Fecal shedding↓; (2) No severe diarrhea; (3) No mortality; (4) No obvious intestinal lesions and no PEDV antigens detected.	Piglets (5 d old, *n* = 5); intranasally twice with 2×10^6^ TCID_50_/pig.	Piglets (33 d old, *n* = 5); 2 × 10^7^ TCID_50_/pig with AH2012/12. (1) Fecal shedding↓; (2) Fecal scores↓; (3) Neutralizing antibodies↑; (4) Viral antigens in epithelial cells↓; (5) Virus loads in jejunum↓.	[51]
N93/95A	N^93^A/N^95^A in nsp1	Gn piglets (5 d old, *n* = 4); 100 TCID_50_/pig. (1) Mortality↓; (2) Severe diarrhea rate↓; (3) Fecal shedding↓; (4) Intestinal VH/CD ratios↑.	Gnotobiotic (Gn) piglets (5 d old, *n* = 4); orally with 100 TCID_50_/pig.	Piglets (27 d old, *n* = 3); 10^6^ PFU/pig with icPC22A. (1) No mortality; (2) No severe diarrhea; (3) Fecal shedding↓.	[153]
rPEDV-Δnsp2	Δnsp2	Piglets (3–5 d old, *n* = 27); 2 × 10^5^ TCID_50_/pig. (1) No severe diarrhea; (2) No mortality; (3) No obvious intestinal lesions; (4) Intestinal virus and fecal shedding↓; (5) Viral antigens in epithelial cells↓; (6) Intestinal VH/CD ratios↑;	/	/	[83]
IcPEDV-EnUmt	H^226^A in nsp15	Piglets (7 d old, *n* = 8); 10^5^ TCID_50_/pig. (1) Fecal shedding↓; (2) No mortality.	/	/	[108]
KDKE^4A^	K^45^A/D^129^A/K^169^A/E^202^A in nsp16	Gn piglets (4 d old, *n* = 8); 100 PFU/pig. (1) Mortality↓; (2) Fecal shedding↓; (3) Intestinal VH/CD ratios↑.	Gn piglets (4 d old, n = 8); orally with 100 PFU/pig.	Piglets (25 d old, *n* = 5); 10^6^ PFU/pig with icPC22A. (1) No mortality; (2) Diarrhea↓; (3) Fecal shedding↓.	[113]
KDKE^4A^-SYA	K^45^A/D^129^A/K^169^A/E^202^A in nsp16 and Y^1378^A in S	Gn piglets (4 d old, *n* = 7); 100 PFU/pig. (1) No mortality; (2) Severe diarrhea↓; (3) Fecal shedding↓; (4) Intestinal VH/CD ratios↑.	Gn piglets (4 d old, n = 7); orally with 100 PFU/pig.	Piglets (25 d old, *n* = 5); 10^6^ PFU/pig with icPC22A. (1) No mortality; (2) No diarrhea; (3) Fecal shedding↓.	[113]
icPEDV-mut4	F^44^A of nsp1, H^226^A and H^224^A in nsp15, and D^129^A in nsp16	Piglets (3–6 d old, *n* = 13); 500 TCID_50_/pig. (1) No sign of disease; (2) Fecal shedding↓; (3) No significant intestinal lesions; (4) No antigens detected by IHC.	/	/	[80]
rPEDV-E_Δaa23–aa29_	Δ7 (23–29 aa) in E	Piglets (2 d old, *n* = 7); 10^6^ TCID_50_/pig. (1) Fecal shedding↓; (2) Severe diarrhea↓; (3) Mortality↓; (4) Intestinal lesions and antigens↓.	Piglets (2 d old, n = 7); orally with 10^6^ TCID_50_/pig.	Piglets (23 d old, *n* = 5); 5 × 10^6^ TCID_50_/pig with rPEDV-E_wt_. (1) No mortality; (2) Severe diarrhea↓; (3) Fecal virus shedding.	[57]
RMT	Remolded transcriptional regulatory sequences–core sequences (TRS-CSs) and ΔORF3	Gn piglets (5 d old, *n* = 4); 100 PFU_0_/pig. (1) No mortality; (2) Severe diarrhea rate↓; (3) Fecal shedding↓.	Gn piglets (5 d old, n = 4); orally with 100 PFU/pig.	Piglets (24 d old, *n* = 4); 10^6^ PFU/pig with icPC22A. (1) No mortality; (2) No severe diarrhea; (3) Fecal shedding↓.	[11]

Note: ^1^ ↓, decreased; ↑, increased; / means not suitable or unknown.

## Data Availability

Not applicable.
